# A Narrative Literature Review on Sepsis: A Primary Manifestation of Colorectal Neoplasm

**DOI:** 10.7759/cureus.44803

**Published:** 2023-09-06

**Authors:** Lalain Masood, Agustina Müller, Nayab Z Ali, Anvitha Mummadisetty, Anam Yahya, Sai Shivani Burugu, Rabia Sajid, Mohit Lakkimsetti, Sowmya Sagireddy, Zain U Abdin, Zahra Nazir

**Affiliations:** 1 Department of Internal Medicine, Bahria University Health Sciences Campus, Karachi, PAK; 2 Department of General Medicine, Austral University Hospital, Pilar, ARG; 3 Department of Internal Medicine, Sialkot Medical College, Sialkot, PAK; 4 Department of Internal Medicine, Modern Government Maternity Hospital, Hyderabad, IND; 5 Department of Pharmacology, Dr. D. Y. Patil Medical College, Navi Mumbai, IND; 6 Department of Psychiatry, Hereford County Hospital, Hereford, GBR; 7 Department of Internal Medicine, Mayo Hospital, Lahore, PAK; 8 Department of Internal Medicine, Mamata Medical College, Khammam, IND; 9 Department of Internal Medicine, Coney Island Hospital, New York, USA; 10 Department of Internal Medicine, District Head Quarter Hospital, Faisalabad, PAK; 11 Department of Internal Medicine, Combined Military Hospital, Quetta, PAK

**Keywords:** difficult-to-treat pathogens, microbial association, complication, colorectal cancer (crc), abdominal sepsis

## Abstract

Sepsis and colorectal cancer (CRC) exhibit a complex relationship that warrants further exploration. This review delves into the interplay of factors between sepsis and CRC, uncovering shared pathophysiological traits and potential bacterial associations. Understanding these connections could pave the way for earlier diagnosis, improved management, and enhanced outcomes in CRC patients. The role of immune system dysfunction, hypoalbuminemia, and specific microbial imbalances, such as Streptococcus bovis and Clostridium septicum, are discussed. Recognizing sepsis in CRC patients is crucial for timely intervention, and tailored approaches encompassing antibiotic therapy, source control measures, and cancer treatment are essential for comprehensive care. Monitoring biomarkers and ratios can provide valuable insights into complications and overall health outcomes. A multidisciplinary approach involving various specialists is necessary to address the global burden of CRC and its association with sepsis while exploring novel interventions, such as fecal microbiota transplantation and personalized care. We conducted a thorough search using reputable databases such as PubMed, Scopus, and Google Scholar to investigate the connection between sepsis and CRC. We refined our search terms, utilized sidebar filters, and examined references in selected articles. This meticulous process helped us create a comprehensive literature review and gain valuable insights into this relationship.

## Introduction and background

As the fourth most significant cause of death, colorectal cancer (CRC) is the second most prevalent adult cancer in women and the third most prevalent in men, accounting for 9.2% of fatalities globally [1]. Colorectal neoplasms are heterogenetic and sporadic [2]. Concerning the various mechanisms playing a role in carcinogenesis, the Knudson two-hit hypothesis is an old but golden rule that addresses this question. Accordingly, although host factors play a significant role, the second hit from various environmental factors is the one that aggravates carcinogenesis [3]. Interestingly, an alteration in the gut microbiota with harmful bacteria and viruses has been associated with developing colorectal neoplasm [4,5], including Streptococcus bovis, Streptococcus gallolyticus, Helicobacter pylori, Bacteroides fragilis, Enterococcus faecalis, Clostridium septicum, Fusobacterium spp., and Escherichia coli [6]. However, factors leading to dysbiosis, whether as a cause or a consequence of CRC, must be explored [7]. 

Though there are many ways a colorectal neoplasm can present (e.g., changes in bowel habits, rectal bleeding, anemia in case of occult bleeding, discomfort in the stomach, and unexplained weight loss), bacteremia with the organisms mentioned above is the sign currently explored [8-11]. Sepsis may be the first symptom in a few percent of patients, causing diagnostic confusion and delaying management; however, there are likely many factors in the complex link between colon cancer and sepsis. Many pathophysiological hallmarks of sepsis and cancer can be traced back to the host’s immune system’s inability to respond effectively to the initial event of an insult [12]. An inflammatory cascade is due to tumor-related events, such as necrosis, intestinal perforation, and bone metastases. 

The term “sepsis” refers to a potentially fatal organ dysfunction brought on by an exaggerated host response to infection [1]. Over five million fatalities a year can be linked to sepsis, with an annual incidence of approximately 20 million cases and a death rate of approximately 26% [13]. Sepsis is usually associated with bacterial or fungal infections that can develop anywhere, including the respiratory, abdominal, and genitourinary systems. Patients with weaker immune systems, those who are very young or very old, and those with coexisting chronic diseases (e.g., respiratory disorders, heart conditions, diabetes, cancer, and kidney disease) are all at a higher risk for developing sepsis [13]. This association highlights that patients with colon cancer have weakened immune systems, so this tumor-induced immunosuppression may make them more vulnerable to sepsis and the related consequences. According to prior studies, people with cancer have a nearly 10-fold higher risk of developing sepsis than people without cancer [14].

Furthermore, this patient cohort has a 30% higher mortality risk than other patients with critical sepsis [14]. Therefore, a compromised immune system can make a person susceptible to various pathogens. Hence, the peculiar infections caused by Clostridium septicum and Enterococcus [15,16] have been associated with colorectal carcinoma. Currently, nonconclusive evidence in the medical literature supports a causal relationship between bacteria and CRC. 

Therefore, due to shared pathophysiological traits brought on by the host’s immune system’s inability to handle an initial trigger, the possibility of their interrelated outcomes is a definite cause for concern. Hence, exploring the interdependent relationship between sepsis and cancer can pave the way for groundbreaking research avenues, potentially enhancing cancer outcomes by regulating the faulty immune system responsible for sepsis [17]. Fundamental and clinical research findings point to hypoalbuminemia due to the body’s response to cancer [18]. Moreover, this low albumin level renders the patient more susceptible to infection [19]. In addition, depending on the microbe, different associations have been made with various types of CRCs and the part of the colon most affected [20-22]. 

As discussed above, a careful understanding of unique presentations, such as sepsis, is necessary for early diagnosis and treatment because CRC incidence and mortality are high. This study aims to undertake a comprehensive literature review on sepsis as a primary sign of CRC, expecting that a deeper understanding of the underlying mechanisms may lead to better patient outcomes due to earlier diagnosis and more effective management.

## Review

Pathogenesis and molecular mechanisms of sepsis and colorectal neoplasm

Sepsis can cause widespread inflammation, tissue damage, organ dysfunction, and potential fatality if not promptly treated. With CRC, there is a risk of severe abdominal infection and sepsis. CRC, combined with gastrointestinal perforation, is a common and critical disease that, if not properly managed, can progress to sepsis, multiple organ failure, and death [23]. Independent risk factors for sepsis in CRC patients with gastrointestinal perforation include anemia, intestinal obstruction, preoperative chemotherapy, acidosis, and low albumin levels. Perforation of the digestive tract can lead to intra-abdominal infection, peritonitis, abscess formation, and the progression to sepsis and septic shock. The combination of CRC and gastrointestinal perforation has a poor prognosis, so a reliable model can help detect patients at high risk of developing sepsis [24]. Digestive tract tumors, including CRC, can contribute to developing anemia through multiple mechanisms. These tumors can secrete cytokines that inhibit the normal function of hematopoiesis, lead to malnutrition, cause deficiencies in essential nutrients (e.g., vitamins, iron, and folic acid) necessary for healthy blood cell production, and cause gastrointestinal bleeding, resulting in blood loss and further exacerbating anemia. As a result, anemia is more commonly observed in patients with CRC, particularly those in the middle and advanced stages [25-27]. Patients with anemia often experience decreased immune function, making them more susceptible to developing sepsis [28-30].

Developing an intestinal obstruction in CRC can lead to complications, including sepsis. When the tumor obstructs the intestinal lumen, it can cause intestinal edema, resulting in increased intestinal permeability, disturbances in water-electrolyte balance, and displacement of intestinal flora. These factors can contribute to the development of sepsis. Patients undergoing preoperative chemotherapy may also experience increased intestinal permeability, further exacerbating the risk of infection and sepsis. Additionally, chemotherapy can suppress the body’s immune system, making patients more susceptible to infections and increasing the likelihood of infection spread [23]. 

Acidosis, characterized by an accumulation of acidic substances and increased hydrogen ion concentration in the blood and tissues, reflects tissue ischemia and inadequate tissue perfusion. If acidosis is not promptly corrected, it can promote the progression of sepsis. Albumin, a protein found in the blood, is essential in maintaining hemodynamic stability, reducing tissue edema, and supporting the body’s immune function. With low albumin levels, its effects are limited, so patients become more prone to sepsis. Therefore, supplementing albumin in patients with gastrointestinal perforation and low albumin levels can be beneficial in reducing the risk of sepsis [25]. 

While postoperative sepsis following CRC procedures is often observed in patients over 65 and those with higher ASA scores, the present study focusing on patients with gastrointestinal perforation may not have yielded similar results. This discrepancy may be attributed to the specific patient population included in the study. Overall, considering these factors and closely monitoring CRC patients is essential, particularly those with gastrointestinal perforation, to avoid the development of sepsis and prevent and treat infections promptly [24]. 

The intestinal immune system helps maintain a balanced environment by controlling cell growth, inflammation, and wound healing. Recent research has linked these immune functions to developing colitis-related CRC. The complex community of microorganisms in the gut, intestinal microbiota, plays a crucial role in this process. Imbalances in the microbiota can promote inflammation and dysplasia in the colon. Specific sensors in our immune system recognize harmful microbial patterns, triggering inflammation and abnormal cell growth. Additionally, certain enzymes in the microbiota can convert dietary substances into active forms contributing to the development of abnormal cells [31].

The exact cause of CRC is still unknown, but genetic and environmental factors are believed to contribute to its development. A diet high in processed foods, animal fat, and red meat, lacking in fiber and fruits, and exhibiting sedentary behavior and obesity is thought to alter gut microbiota composition and increase the risk of CRC, especially in developing countries. Dysbiosis, an imbalance in the function and structure of gut microbiota, has been linked to various diseases, including inflammatory bowel disease (IBD), obesity, colitis, and CRC [31].

Several studies have identified bacterial species associated with the development of CRC, and recent research has highlighted how gut microbiota participates in its progression. The intestinal microbiota can contribute to carcinogenesis by producing secondary metabolites that damage DNA, such as reactive oxygen intermediates, or by directly affecting cell transformation by producing genotoxins. Various bacterial species (e.g., B. fragilis, C. septicum, E. faecalis, H. pylori, S. bovis, E. coli, and Fusobacterium spp.) are believed to play a role in colorectal carcinogenesis. Therefore, studies have provided evidence that changes in the structure of gut microbiota can trigger an immune response and play a crucial role in the epigenetic mechanisms of the host’s intestine [32].

Substantial evidence supports a link between infectious agents and cancers, including CRC [33]. Certain bacterial species associated with the mucosa play a role in the pathogenesis of CRC. Furthermore, specific strains of Enterobacteria have been found to produce genotoxins that can damage DNA [34]. Bacteria may contribute to the accumulation of mutations observed in the progression from adenoma to carcinoma in CRC [35].

Postoperative systemic inflammatory response syndrome (SIRS) and sepsis following surgery for intestinal obstruction are associated with a significant increase in the mortality rate within 30 days. Certain factors have been identified as independent predictors of postoperative SIRS/sepsis, including a high preoperative heart rate, low albumin levels before surgery, and intensive care unit (ICU) admission after the operation. Identifying these modifiable risk factors may reduce post-surgical complications and mortality rates. Additionally, stratifying patients based on these risk factors can aid their management. This approach can help accurately predict and evaluate ICU duration, hospital length of stay, economic costs, and clinical outcomes [36].

CRC can give rise to abscesses, often forming retroperitoneal abscesses when colonic perforation occurs. Fusobacterium nucleatum, an overexpressed bacterium in adenoma and carcinoma tissues, has been associated with CRC development. Experimental studies have shown that F. nucleatum infection promotes the formation of adenomas and carcinomas by generating reactive oxygen species, causing DNA damage and genetic/epigenetic changes. Additionally, F. nucleatum activates specific signaling pathways and toll-like receptors, leading to increased proliferation of CRC cells. Thus, detecting F. nucleatum in patients may have diagnostic potential, as it triggers the production of anti F. nucleatum antibodies in individuals with CRC [32]. 

Studies have also found that F. nucleatum in intestinal infections is a valuable marker for CRC diagnosis. Other bacteria, such as Campylobacter jejuni and Salmonella species, have also been implicated in promoting intestinal inflammation and contributing to developing CRC through various mechanisms. Severe salmonellosis, particularly Salmonella enteritidis infection, is associated with an increased risk of colon cancer development, suggesting a potential contribution of this foodborne infection to CRC. Notably, further research is ongoing to better understand these associations and their potential diagnostic and therapeutic implications [37].

Neutrophils are essential for fighting infections but can cause tissue damage during sepsis. Hence, understanding how immune cells are affected during sepsis is crucial [38-40]. The preoperative neutrophil-to-lymphocyte ratio could be a new way to predict postoperative complications, similar to preoperative albumin levels [41]. Indeed, a slight decrease in serum albumin significantly impacts hospital stay length, surgical site infections, and other health issues [42]. Moreover, antibody levels against Salmonella ser. Typhimurium is higher in colorectal tumor cases than controls, possibly influenced by smoking and dietary iron [43].

Chronic inflammation plays a significant role in CRC development. Bacteria such as S. gallolyticus and F. nucleatum can stimulate the production of inflammatory chemicals, promoting tumor growth. Bacteremia caused by CRC-associated bacteria can be an early sign of CRC, highlighting the importance of colonoscopy for early detection and lower mortality. Conventional culture-based systems are still the standard for detecting bacteria or fungi in sepsis. Blood cultures are positive in about 30-40% of severe sepsis and septic shock cases, and bloodstream infections are often intermittent with low levels of microbes [6].

A multiplex approach has been used to detect S. bovis infection and its association with CRC by measuring antibodies against pilus proteins. Studies on the association between CRC and H. pylori infection have produced different conclusions, even using the same detection methods [44]. New screening tests for CRC use advancements in molecular biology, genetics, and sequencing technologies. These include blood-based tests using circulating tumor DNA and serum proteins, stool DNA or mRNA tests, and methods for analyzing the gut microbiome [45].

Colorectal neoplasm classification and staging 

The third most common cancer worldwide is CRC, the leading cause of morbidity and death among gastrointestinal tumors, ranking fourth after lung, breast, and ovarian cancers [46]. Despite a continuous refinement of the T (tumor), N (node), and M (metastasis) staging system to express disease extent and define prognosis, and eventually to guide treatment, the outcome of patients with CRC may vary considerably even within the same tumor stage. Therefore, the need for new factors, either morphologic or molecular, to more precisely stratify patients into different risk categories is warranted [38]. Survival is stage-dependent mainly, guided by the tumor-node-metastases (TNM) system for TNM assessment. Histopathological evaluation, including assessment of lymph node status, is essential for correct TNM staging [46].

The goal of this staging has been to provide physicians and others with a practical methodology to plan treatment, project prognosis, and measure outcome results. Until recent years, this system has only incorporated elements of the anatomic extent of the tumors determined by clinical and pathologic methods. However, an increasing number of nonanatomic cancer prognostic factors have been identified and studied. These factors are currently used for outcome predictions and treatment decisions [39].

CRC management highly relies on the TNM staging system. Tumor deposits (TDs), important histoprognostic factors, are detected in approximately 20% of CRCs and are associated with poor prognosis. Integration of TDs in the TNM staging remains a subject of lively debate and differs over the successive TNM classifications. Currently, TDs, whatever their number, are considered in pathologic staging only in the absence of lymph node metastasis. However, the medical community is divided over integrating TDs into the TNM staging system [47].

Experimental data support the concept of immunosurveillance of cancer in mice. In humans, the presence of tumor-infiltrating lymphocytes indicates a favorable prognosis. In CRC, the absence of early signs of metastasis (tumor emboli) correlates with a high density of intratumoral effector memory T cells. Furthermore, the time to recurrence and overall survival strongly correlates with the in situ adaptive immune reaction [48,49].

Mechanisms and factors linking sepsis and colorectal neoplasm 

Inflammatory cells, cytokines, and chemokines are present in tumors, while overexpression of cytokines and chemokines can also present. The exact molecular targets and similar pathways are activated or shut down in inflammation and carcinogenesis. Hence, chronic inflammatory states increase the risk of numerous cancers [50]. Chronic inflammation includes IBDs like ulcerative colitis (UC) and Crohn’s disease (CD). Infections in the colorectal area include severe diverticulitis and abscess [51]. 

Chronic inflammation can cause many diseases, including cancer [52]. IBD, including UC and CD, can cause the development of CRC. Many cytokines produced primarily by the gut immune cells, either during or in response to localized inflammation in the colon and rectum, stimulate chronic inflammation, and genetic and epigenetic changes have been shown to lead to the development and progression of CRC [52-57]. The bacterial species identified and suspected to play a role in CRCs are S. bovis, H. pylori, B. fragilis, E. faecalis, C. septicum, Fusobacterium spp., and E. coli. The potential pro-carcinogenic effects of these bacteria, such as genotoxicity and other virulence factors, inflammation, host defense modulation, bacterial-derived metabolism, oxidative stress, and anti-oxidative defense modulation, have been linked to CRC [11,58].

Moreover, smoking and high red meat intake have been associated with CRC risk. Increased iron exposure may be a common factor, favoring the colonization of specific bacterial pathogens that preferentially grow in an iron-rich luminal environment [59].

The gut microbiota inhabits the human digestive system, living in symbiosis with the host. Dysbiosis, an imbalance between the beneficial and opportunistic gut microbiota, is associated with several gastrointestinal disorders, such as irritable bowel syndrome and IBD, represented by UC, CD, and CRC [7]. Dysbiosis can disrupt the mucosal barrier, resulting in the perpetuation of inflammation and carcinogenesis. The increase in specific groups of harmful bacteria has been associated with chronic tissue inflammation and the release of pro-inflammatory and carcinogenic mediators, increasing the chance of developing CRC following the inflammation-dysplasia-cancer sequence in IBD patients [7,60].

Post-op complications following colorectal surgeries: Anastomotic leak, a post-op complication, can lead to further infection and, ultimately, sepsis, delaying treatment. Other treatment modalities such as anterior resection, abdominoperineal resection, hemicolectomy, and chemotherapy can also lead to sepsis and further complications. The pathologic state of immune suppression in sepsis is associated with the subsequent development of ICU-acquired nosocomial infections such as ventilator-associated pneumonia, urinary tract infection, catheter-associated bacteremia, antibiotic-associated diarrhea, and Clostridium difficile enterocolitis [61].

Immunosuppression: Sepsis can lead to immune system dysfunctions and contribute to the development and progression of colorectal neoplasms. For example, the human polyomavirus JC virus is a small double-stranded linear DNA virus. The JC virus is present in the kidneys in 80% of the population and remains latent in healthy individuals. In immune-compromised hosts (e.g., organ transplantation recipient or HIV seropositivity), it becomes reactivated. Its genome encodes for the large T-antigen protein that can interact with p53 and pRB (tumor-suppressor proteins) and other key signaling pathways [62-63].

Environmental and modifiable factors (e.g., smoking, alcohol, obesity, unhealthful dietary habits, diabetes, and physical inactivity) are CRC risk factors [64]. Obesity is a meaningful contributor to CRC and a poor prognosis factor in cancer development. Obesity has been associated with several obesity-related cancers, including CRC [65]. Obesity initiates different cellular and molecular pathways, eventually forming tumors. Adipose tissue produces many hormones and pro-inflammatory cytokines, such as interleukin 6, tumor necrosing factor-α, leptin, and adiponectin provide desirable inflammatory microenvironment conditions for cancerous cells. Current studies have revealed that adipose tissue stimulates proliferation, migration, angiogenesis, and oxidative stress induction [65].

Moreover, visceral fat (abdominal fat) is associated with insulin impairment and high IGF2 serum levels. This cohort study showed that adolescents (male and female) with overweight or obesity conditions were prone to colon and rectal cancer. Adipose tissue assumedly produces different hormones and pro-inflammatory cytokines, including IL-6, TNF-α, leptin, and adiponectin, which could provide desirable micro-environmental inflammatory conditions for cancerous cells. Furthermore, high levels of IL-23 and IL-10 in serum and IL-8 and IL-6 in the microenvironment are associated with the progression of CRC [64-65].

Studies have demonstrated that insulin activates the phosphoinositide 3-kinase-protein kinase B-mammalian target of rapamycin (PI3K-Akt-mTOR) and RAS-mitogen-activated protein kinase (RAS-to-MAPK) pathways by binding to the cognate receptor or insulin-like growth factor receptor, which in turn can lead to downstream cellular proliferation and protein synthesis in tumor cells. For example, rat models have demonstrated that insulin can induce the proliferation of colorectal epithelial cells and the development of aberrant crypt foci, the primary neoplastic lesions in colorectal development. In colonic tumor cells, the expression of the insulin receptor protein is elevated, particularly isoform A, exerting mitogenic effects [64-65].

Gut microbiota and CRC

Intestinal floras have taken an imperative role over the last 10 years, as their extension and exclusive variety have attracted the attention of many scientists. It is believed that more than 10 of 14 microorganisms form them, a surprising number exceeding 10 times the totality of human cells and more than 100 times the genomic content we possess [66]. These impressive results were recently reviewed, where the human cell-to-bacteria ratio was believed to be closer to 1:1 [67]. Its delicate balance between bacteria, archaea, eukaryotes (e.g., yeasts and other fungi), and viruses is necessary to perform its functions properly [68]. Among those highlighted are glycans processing as an energy source, the production of essential vitamins (e.g., K and B12), the immune function against pathogens, and the regulation of the host’s general immunity [66]. 

The symbiosis generated between the host’s immune system and the contents of the gastrointestinal tract is regulated by various components that fulfill the barrier function physically through the epithelium and its adjacent mucosa and molecularly through enzymes, other antimicrobial components such as exotoxins, IgA, and other components of the regional immune system [55]. The extent of environmental factors influencing the imbalance of the mentioned flora are well known, such as diet variations, antacid and laxative use, and antibiotic abuse [69]. They threaten the integrity of the barrier, its functions, and the host’s health, making it more likely to develop dehydration symptoms secondary to diarrhea and vomiting typical of the previously mentioned imbalance or severe systemic infectious symptoms due to permeability, which could lead to the translocation of components of a dysregulated microbiome [70].

An association is beginning to emphasize the importance of the balance of the intestinal flora to prevent cancer via various mechanisms by which this immune balance would regulate inflammation generated by organisms (e.g., B. fragilis, Clostridioides and Clostridium spp., E. faecalis, E. coli, F. nucleatum, H. pylori, Peptostreptococcus anaerobes, the S. bovis group, and sulfate-reducing bacteria) to keep the antitumoral function intact [67,71]. However, this prevention is developed around the ability to regulate potential pathogens’ activity, the study of microbiomes and their variety, and the ability of microorganisms to process different foods, highlight fiber, and obtain metabolisms that could act in chemoprevention [68]. Therefore, pathogenic overgrowth and poor diet are the measures we most want to develop in this study while considering radiation, smoking, and alcohol consumption among the external causes promoting DNA mutations that hinder the modulation of proto-oncogenes and tumor-suppressor genes, stimulating the tumor’s free growth and progression [72].

Hence, the translocation of these organisms into the circulation is generated from barrier transgression, either by injury or the permeability generated by inflammation. Thus, it is logical that, if a patient presents with a septic condition produced by organisms that should be found almost exclusively in the intestinal lumen, it should be standard to rule out the existence of CRC simultaneously [73]. Numerous studies have pointed to a relatively short list of microorganisms as CRC markers (e.g., S. bovis, S. gallolyticus, C. septicum, and the entire Enterococci family) [9,11,16,70,74]. The recommendation is to continue with the pertinent studies to rule out possible colorectal pathology [75,76].

Increased diagnosis of CRC in patients with bacteremia 

The rectum is the body part with significant septicity because bacteria such as Enterococcus, Pseudomonas, and Serratia are present. Therefore, when anastomosis is performed, the possibility of septicity increases [77]. Moreover, an increased frequency of FDG deposits was found in the Streptococcus genus subgroup [78]. Within the BSI group, 42% of colorectal lesions included one hostile, E. faecalis, precancerous S. gallolyticus and E. faecalis, and benign E. faecalis and E. coli [78]. Pro-inflammatory cytokines increase vascular permeability, making it easier for the bacteria to enter the blood circulation and progress to produce premalignant injuries, resulting in sepsis later on [16,78].

S. bovis and its subclasses, types I and II, have been linked to endocarditis, colorectal carcinoma, and biliary tract illnesses [10,75,78,79]. The relationship between S. bovis and colorectal carcinoma has been well acknowledged recently, mainly including S. gallolyticus bacteria [10,37,71,75,79,80]. However, whether the bacteria causes colon cancer or the cancerous lesions cause favorable conditions and a conduit for bacteria to multiply and enter the blood circulation is ambiguous [79]. Other studies have observed a well-established correlation between S. bovis genus and CRC [71].

However, another study revealed that not all subgroups of S. bovis are correlated with colonic neoplasia [10]. In the digestive tract of healthy people, S. bovis ranges from 15% to 62% [79]. In 2015, Glibetic et al. [79] proposed that S. bovis is a driver and a passenger cancer microorganism [79,80]. This microbiota is a contributory element that expedites the development of CRC [10,37,71,79,80]. In the presence of S. bovis septicemia, there was a seven-fold increase in the possibility of having CRC, as reported in the study [9,75]. As published by Glibetic et al., a patient transfused with platelets went into septic shock because the platelets were contaminated by the S. bovis species [79].

Moreover, the Streptococcus genus has been categorized into seven subgroups, including S. gallolyticus subgroup gallolyticus, S. gallolyticus subgroup macedonicus, S. gallolyticus subgroup pasteurianus, S. infantarius subgroup infantries, S. lutetiensis, S. elastolytic, and S. equinus [71]. Using in vitro cell cultures and mice models of CRC, Macha et al. showed that S. gallolyticus actively promotes colon cancer cell proliferation and tumor progression, indicating that its presence in CRC is causative, not only temporary or symbiotic. Hence, S. gallolyticus serology may be used as a new marker for the risk of developing CRC [72].

Another anaerobic, Gram-positive bacteria found in the intestine and linked with primary malignancy in more than 80% of cases are C. septicum [72]. Patients infected with C. septicum had digestive tract illnesses and CRC [71]. It has a strong association with CRC and immunosuppression. C. septicum is linked with an increased death rate that can occur within 24 hours if the disease is not detected earlier and prompt treatment is not given instantly [9,72]. It has been hypothesized that the low-oxygen level and acidic microenvironment of aggressive tumors promote the growth of C. septicum spores through anaerobic glycolysis, demonstrating that C. septicum has a non-causative correlation [72]. C. septicum spores may invade blood circulation through perforations in the digestive tract or colorectal epithelium, causing sepsis [9,16,72].

Moreover, other Clostridia species correlate with CRC [71,80]. For instance, Xu et al. [49] studied an increase in the amount of Clostridioides difficile in patients of CRC who were not operated on, so C. difficile was not a hospital-acquired microorganism. Another study suggested that the frequency of C. difficile in malignant tissues was 60%, while in healthy tissues was 20%. In addition, C. difficile antibodies were found persistently in CRC patients (i.e., 66.7% compared to clinically healthy individuals at 30.8%) [71]. Hence, C. difficile is also associated with CRC [37]. C. septicum has four main exotoxins (i.e., α-, β-, γ- and δ-toxin), which lead to myonecrosis. After C. perfringens and C. tertium, C. septicum is the third most prevailing bacteria that causes sepsis [74-76].

Clinically, S. gallolyticus is correlated to early-stage neoplasms, whereas C. septicum bacteria-correlated cancers are mainly advanced stage (i.e., 58% in stages III and IV), with an average size of 7 cm compared to S. gallolyticus’ average size of 1.5 cm, indicating that S. gallolyticus appears in intermediate stages of CRC [80]. In addition, more than two-thirds of CRC patients bear the colibactin-producing E. coli genus in their digestive tract, and the figure of carriers is increasing in the Western world. Because of their capacity to produce the bacterial cytotoxin colibactin, polyketide synthase (pks) genetic island-positive strains of calmodulin-positive B2 E. coli (pks + E. coli), commonly known as B2 E. coli, are particularly cytotoxic and have been linked to CRC [72]. Moreover, E. coli acts as a passenger and driver microorganism [10].

F. nucleatum is a Gram-negative, obligate anaerobe that is opportunistic and frequently found in the oral cavity. Recently, F. nucleatum has been accounted for inside the essential sore site of the cecum and the rectum in patients with malignant colon growth [6,71,72,80]. Furthermore, F. nucleatum is described as a passenger and driver microorganism [9,10]. Overexpression of miR-135b as an inflammatory relation has been linked with F. nucleatum in individuals with CRC [72].

Salmonella enterica, a Gram-negative microorganism, correlates with CRC through its proteins, typhoid toxin, calmodulin, and AvrA [72]. Furthermore, an anaerobic, Gram-negative, bile-resistant microorganism known as B. fragilis is the strain of enterotoxigenic B. fragilis (ETBF), which strongly correlates with CRC [6,80-82]. The ETBF and pks+ E.coli are alpha bugs (i.e., act as drivers and passengers in cancer progression) [9,10,80]. An enormous amount of bacteria is found in the colon, resulting in more than 1,011 organisms per gram of wet weight, with most as anaerobes, including 25% of bacteroides [82,83].

Moreover, E. faecalis, a Gram-positive, facultative anaerobe, catalase-negative bacteria, has an essential correlation with CRC. However, various studies have diverse views regarding this link [16,37,72]. Many studies have suggested an association between E. faecalis and CRC [72,80]. Another study suggested that, in the fecal flora of patients with CRC, was an increase in the levels of E. faecalis compared to patients with polyps and healthy individuals [71]. One study also suggested a symbiotic correlation between E. faecalis and colon cancer [16].

However, one study suggested that the direct cause of gastric cancer is H. pylori, labeled as a carcinogen of the digestive tract by the International Agency for Research on Cancer. Infected patients have 1.4-fold higher possibility of developing CRC than uninfected individuals [71]. H. pylori has a carcinogenic role at the preliminary stage of malignant tumor formation. In one meta-analysis of studies, 17,416 patients with CRC and 55,811 controls indicated that H. pylori contamination was related to a higher possibility of developing CRC (OR = 1.70) [71]. Another study termed H. pylori as alpha bugs (i.e., bacterial drivers) [9].

Moreover, post-streptococcus anaerobic levels were high in fecal samples and the gut mucosa of patients with CRC compared to the control group, as revealed by a study [71]. Furthermore, microorganisms were reduced in ApcMin/+ mice animal models of unconstrained CRC. The experimental group received P. anaerobic for 10 days, while the control group received E. coli strain MG1655. Further, the study revealed that P. anaerobic predominantly colonized the colon and was frequently found in the foci of intestinal dysplasia [71].

In addition, another anaerobic microorganism, sulfate-reducing bacteria, can be a factor in developing CRC and was more present in increased levels in stool samples of CRC patients compared to healthy individuals. Furthermore, there is a higher possibility of developing CRC if one’s diet is rich in sulfur [71]. Lactobacillus bacteria is another correlated with diseases such as ischemic colitis, cancer, and IBD [82]. Klebsiella species endophthalmitis has also been described as an indicator of unrecognizable CRC in Chinese patients, showing that CRC can also present through uncommon infections that barely correlate directly with an underlying malignancy (e.g., skin infection) [37]. Table 1 summarizes the correlation of various bacteria in CRC.

**Table 1:** Correlation of bacteria in colorectal cancer [10,71,72,78]

BACTERIA	PATHOGENESIS
Streptococcus bovis (Subtype Gallolyticus)	Inflammatory cytokines (IL-1β, TNF-α, IL-6, and IL-8) produce nitric oxide, superoxide, peroxynitrites, and hydroxyl radicals, altering the DNA structure and leading toward neoplasm. Cellular proliferation occurs with the upregulation of β-catenin, c-myc, and cyclin D.
E. coli	Colibactin production leads to epithelium damage.
F. nucleatum	The binding of FadA to E-cadherin impedes the activity of the tumor-suppressor gene, leading to transcription. Myc and Cyclin D1 result in increased cell multiplication, damaging the DNA, while many CRC cells are multiplied.
Salmonella enterica	The WNT/β-catenin pathway is activated by AvrA, also enhancing the STAT3 signaling pathway with P53 as its target, leading to CRC. High levels of IL-22 impede apoptosis and stimulate tumor growth.
ETBF	Transcription and translation of proto-oncogene c-myc occur in CRC cells, resulting in cell proliferation.
C. septicum	Through anaerobic glycolysis, the acidic, low-oxygen microenvironment of aggressive tumors enhances the growth of C. septicum spores.
E. faecalis	The ANGPTL4/FIAF gene is downregulated, triggering macrophages and initiating chromosomal instability in primary epithelial cells. These bacteria also produce ROS and extracellular superoxide, damaging the colon’s DNA and progressing to cancer.
H. pylori	Mucosal damage commences when H. pylori LPS binds to TLR4 on the mononuclear cell surface, leading to chronic inflammation and generating pro-inflammatory cytokines IL-1β IL-18 and TNF-α, finally leading to Th1 and Th17 cell differentiation.
Poststreptococcus anaerobius	This bacterium causes the upregulation of genes responsible for AMPK signaling, TLR signaling, and cholesterol biosynthesis pathways. It also leads to increased multiplication of cancer cells (HT-29 and Caco-2). ROS levels, pro-inflammatory response, cholesterol synthesis, and cell multiplication increase in the colon.
Sulfate-reducing bacteria	These bacteria produce H2S that damages the DNA, causing oral and colon cancer progression through ERK1/2 and Akt pathways. The RAS/MAPK pathway is also involved, leading to cell multiplication and tumor formation.

CRC is the third most prevalent and deadly cancer globally and has caused massive economic consequences [71,80,81,84-87,88]. In 2020, 1.9 million new cases were reported, of which 0.9 million deaths occurred [89,90]. Due to the adoption of Western lifestyles and the gradual aging of the masses, CRC has become widespread [71,80,91,92]. After 50 years of age, CRC is more prevalent in men than women [71]. People at risk have a positive family history of CRC, familial polyposis syndrome, hereditary nonpolyposis, and chronic IBDs. Other factors include smoking, obesity, diabetes, excessive fat and alcohol consumption, sedimentary lifestyle, poor diet, and a diet rich in N-nitrous compounds [10,16,71].

One study reported that, with 1.8 million new cases and nearly 900,000 deaths worldwide, CRC ranks third in incidence and second in death rate, while an additional study suggested that, by 2030, the worldwide burden of CRC would rise to 2.2 million new cases and a 1.1 million yearly mortality rate [85]. However, in recent decades, CRC incidence and mortality among adults over 65 have continuously reduced [81]. Postoperatively, many patients continue to experience infectious complications that, if not detected promptly, can result in sepsis, multiple organ dysfunction, and even death [41,81,84,85,89].

Therefore, to describe the relationship between sepsis and CRC outcomes, Polimeno et al. studied 311 patients undergoing liver resection of colorectal metastases, which revealed that 51% of postoperative disease was because of sepsis [85]. Various studies have also exhibited that bacterial translocation plays a significant role in increasing the incidence of postoperative infections [86]. However, one study observed that comorbidity and female sex who had non-CRC were mainly related to CRC [75].

Another study observed a decrease in the incidence mortality rate of CRC in developed nations due to many factors, including advancements in perioperative procedures, radiotherapy, and chemotherapy [91]. However, a sharp rise in fatalities and incidence of CRC has also been seen in developing countries [91-93]. The study also reported a rise in the early diagnosis of CRC in the US and that the disease is hindered through polypectomy [91]. Another study proposed that CRC incidence and mortality rates vary significantly across HDI levels, with different gradients. Three patterns of CRC incidence and death trends were established in the study. First, the most recent decade has witnessed a rise in incidence and fatality in countries in economic transition (e.g., medium and high HDI countries, such as the Baltics, Russia, China, and Brazil). Second, in very high HDI countries such as Canada, the UK, Denmark, and Singapore, the incidence has risen with a simultaneous decline in deaths. Third, in a number of the highest HDI-indexed countries such as the United States, Japan, and France, there has been a reduction in both incidence and death [92]. However, of 185 nations, 10 had the highest rate of male CRC diagnoses in 2018, while no country had the highest rate of women CRC diagnoses [93].

Sepsis is the leading cause of death in the ICU, resulting in 20% of annual deaths worldwide [82]. Patients with the disease are especially vulnerable to developing it because of immunosuppression, recurrent hospitalizations, intricate medical procedures, and illnesses [82,88]. Studies have also reported that mortality due to sepsis-related multiorgan failure is higher than mortality due to cancer [88]. Cancer-associated sepsis accounts for 8.5% of all cancer deaths in the US annually, and approximately 20% of sepsis survivors develop cancer, indicating a correlation. According to retrospective analyses, cancer is one of the most common comorbidities among patients who develop sepsis, and cancer patients die from sepsis 2.3 times more frequently than cancer-free patients. Moreover, there is a strong link between sepsis and an increased liver, lung, and colon cancer risk [82]. Therefore, tender care should be taken when performing colorectal surgeries because, if a perforation of the colonic mucosa occurs, it can lead to bacteremia, which may result in sepsis and reduce the possibility of survival.

Sepsis as a complication of CRC

Of the various types of colon cancers, it is fascinating that most bacteremia cases uncovered the underlying adenocarcinomas [21,22,94,95]. Hence, dysbiosis promotes the adenoma-carcinoma sequence. Interestingly, sepsis can develop at any stage between the adenoma-carcinoma sequence, depending on the time of involvement of bacteria in the cascade. Though a perfect reason cannot be explained, S. bovis septicemia is usually associated with premalignant lesions or an adenoma (i.e., early involvement) [21,96]. Since progress into an invasive tumor is slow, early treatment can prevent further progression. However, no such relationship has been found with other microbes. Furthermore, various organisms tend to colonize different parts of the colon, which likely decides the affected part. One such association can be made with F. nucleatum, which colonizes the proximal colon and commonly causes adenocarcinoma of the ascending colon [97]. Similarly, most C. septicum bacteremia cases were found to be associated with adenocarcinomas involving distal ileum and cecum [98,99,100].

Indeed, cancer has been implicated in promoting dysbiosis and subsequent bacteremia. Cancer-related tissue damage and epithelial barrier disruption can lead to septicemia [6]. Like all other solid tumors, CRC exhibits “tumor-elicited inflammation.” Though the mechanisms are poorly defined, IL-23 and IL-17 are thought to play a crucial role. Neoplastic cells in colon cancer show an upregulation of IL-23, which is also promoted by microbial products, further enhancing tumor growth, progression, and the production of IL-17 response. Cancer cells also produce defective barrier proteins, which permit the easy entry of microbial products into the tumor and promote its growth. Likewise, they can also gain access into the bloodstream and cause bacteremia [101].

Recent studies have demonstrated that sepsis has long-term patient complications, such as cognitive and functional impairment, prolonged inflammation, and immune dysfunction [102]. Sepsis is said to have an initial hyperinflammatory phase that evolves into an immunosuppressive phase [103,104]. Host immunity in sepsis has several controversial theories that explain the role of innate immunity and adaptive immunity during different stages of sepsis. However, overall, it was concluded that both innate and adaptive immunity cause immunosuppression through several mechanisms, which can further lead to deaths due to secondary bacterial infections [62,105]. Additionally, this finding has opened a gateway regarding immunotherapy in patients with sepsis. Nevertheless, understanding that immunotherapy can be given only during the later stages of the immunosuppressive phase of sepsis is crucial [62].

Another term that needs attention is septic shock. According to the sepsis-3 guidelines, septic shock is persistent hypotension needing vasopressors to sustain a MAP of ≥ 65 mm Hg and with serum lactate counts > 2 mmol/L despite proper volume resuscitation [1]. If sepsis is unrecognized early and not treated promptly, patients can end up in septic shock with multiorgan failure leading to death. Sepsis is responsible for 9% of all cancer-related deaths, and the mortality rate is higher in cancer patients than in non-cancer patients [106,107].  

Moreover, sepsis is associated with a high rate of morbidity and mortality. In May 2017, the World Health Assembly and the World Health Organization declared sepsis a global health priority [108]. However, definitions of sepsis have continually changed. Per the most recent guidelines, sepsis is an increase of ≥ 2 in sequential organ failure score (SOFA) points in patients with infection [1]. Early recognition of sepsis has been associated with improved survival [109,110]. Patients who meet the above criteria should be immediately optimized hemodynamically, with blood samples collected for blood cultures and sensitivity before starting antibiotics. This step guides in understanding the source of the infection, which helps in accurate and complete sepsis treatment while reducing its recurrence rate [111].

In addition, specific associations have been made with colorectal neoplasms after sepsis from specific organisms has been identified. Rather than sepsis directly, varied presentations have been reported, such as surgical site infection with S. bovis, cases of gas gangrene with C. septicum, and many more. Such cases should be promptly identified using the above guidelines before they become sepsis. Simultaneously, infection with such organisms should prompt the screening for colorectal neoplasm if they are an unknown case [112,113].

Current research has promoted the use of biomarkers as a non-invasive method in the early diagnosis of CRC due to the increasing association of dysbiosis with carcinogenesis [114-116]. Such early diagnosis of colorectal neoplasm helps properly treat cancer and halts its progression, otherwise leading to grave complications such as sepsis. Quantification of fecal Fusobacterium has been extensively studied as a biomarker for early recognition of adenoma and CRC. The historically used FIT for cancer screening is considered to have low sensitivity for advanced disease. However, when combined with the above marker, it shows a significant increase in sensitivity to 92.3% and specificity to 93.0% [115]. Not only Fusobacterium but the quantification of many other microbiotas such as Prevotella copri, Gemella morbillorum, Parvimonas micra, Cetobacterium somerae, and Pasteurella stomatitis have helped diagnose colorectal neoplasm [114].

Another biomarker from research is the prevalence of S.bovis/gallolyticus antigens or the antibodies in the serum of patients with occult colorectal neoplasms. The presence of antigens can also be considered silent bacteremia [117]. One study underscored that positive antigen or antibody (IgG) titers can be used as a screening test for underlying neoplasm of the colon [118,119].

Management of sepsis in CRC

Sepsis in CRC patients with CRC must be managed with a holistic approach, incorporating oncologic concepts with intensive care therapies. The critical elements of treating sepsis include promptly initiating appropriate antibiotic medication, fluid support, and hemodynamic preservation. Within the first hour of identifying sepsis, appropriate antimicrobials should be initiated after acquiring relevant samples for culture without considerably prolonging the treatment [120].

The first round of antimicrobials must be broad-spectrum, targeting the potential infection. In more severe situations such as septic shock, opting for a combination of antibiotics is best. Notably, supplementary Gram-negative antibiotics, such as aminoglycosides and fluoroquinolones, should be added in patients at high risk of contracting infection from antibiotic-resistant microbes to increase the likelihood of having at least one efficient antibiotic [121]. The infection source, the patient’s age, prior antibiotic therapy, local guidelines, and immune suppression should be considered when choosing antimicrobial agents [122,123].

These broad-spectrum antibiotics should be halted and replaced with a tailored therapy (either monotherapy or combined medications) once the infectious agent has been confirmed and antibiotic susceptibility has been established [122]. Sepsis in colon cancer patients can be brought on by bacteria such as S. bovis and S. gallolyticus, E. faecalis, C. septicum, Fusobacterium spp., and Escherichia coli, all believed to be linked to CRC [7]. It has long been known that S. gallolyticus is strongly associated with CRC. The minimum inhibitory concentration approach is used in these patients to assess penicillin susceptibility, guiding treatment choices and course length [124].

Hemodynamic implications of sepsis include vasodilation and capillary leak, which reduce blood flow to tissues and ultimately result in organ dysfunction. In sepsis and septic shock cases, resuscitation aims to enhance tissue oxygenation, recover intravascular volume, and rectify organ damage [123]. Within three hours after identifying severe sepsis or septic shock, a 30 mL/kg crystalloid bolus is advised [120].

However, different elements come into play with CRC, such as the necessity of using source control measures such as surgical intervention for bowel perforation or percutaneous drainage in the event of an intra-abdominal infection spread. In cases when tumors are causing obstructions or perforations, prompt treatment is essential to eliminate the root cause of infection and avoid future challenges. Hence, tailoring treatment strategies to each patient’s unique presentation, medical background, and need for immediate care is crucial [125], with surgeons, medical oncologists, interventional endoscopists, and radiologists working together to treat patients when presented with an obstructed or perforated bowel.

In addition, treating the underlying CRC is crucial when treating sepsis. Cancer treatment choices may include surgical resection, chemotherapy, radiation therapy, or a combination, depending on the type, stage, and extent of the cancer [125]. Taking preventative steps is also integral in reducing the likelihood of sepsis since numerous reports of the altered bacterial composition of the gut microbiota have pointed to the critical role of dysbiosis in CRC [7]. By transferring the intestinal microbes from a healthy person through the mouth or the intestines, fecal microbiota transplant (FMT) is the most cutting-edge method for improving the composition of the microbes in one’s gut [126]. Due to the novelty of FMT as a therapeutic approach to altering gut microbiota and its relatively recent use, there need to be more long-term safety trials [127].

Tumors, as shown by Babson et al. [127], capture plasma proteins such as albumin and exploit their breakdown products for growth, resulting in hypoalbuminemia [127]. In addition to predicting infectious consequences in non-infectious conditions, hypoalbuminemia is linked to the onset and severity of many infections [19]. Sepsis in these patients requires individualized care based on the patient’s condition and the medical team’s advice. The most promising results can only be achieved by the combined efforts of multiple medical experts (e.g., surgeons, oncologists, infectious disease specialists, and critical care physicians). In addition, counseling services and palliative care providers can help alleviate the emotional toll of sepsis and a cancer diagnosis, so these steps should not be overlooked.

This literature review assessed records from two large international databases: PubMed and Google Scholar. However, our surveyed databases may not include all reports regarding the relationship between sepsis and CRC since the search was limited to 10 years or fewer from this publication. Studies regarding pediatric and geriatric populations were excluded, as were studies in languages other than English. Despite these limitations, reviewing the current evidence can help other healthcare providers understand sepsis better and take appropriate precautions for patients under their purview. Reports and research on the pathophysiology of sepsis must continue to identify strategies to combat the incidence of CRC and tailor its management per patient.

## Conclusions

This review of the recent literature shows that bacteremia is strongly associated with the development of colorectal neoplasm, CRC, and sepsis. Numerous bacteria are involved in developing CRC, leading to septicity. Sepsis, as the primary manifestation of CRC, has increased the death rate compared to cancer alone. Moreover, various other reasons have led to increased CRC incidence, which may be prevented by taking measures such as an active lifestyle, healthy dietary options, and delicate caring for intricate gut-related surgical procedures.
